# Competition between Direct Detection Mechanisms in Planar Bow-Tie Microwave Diodes on the Base of InAlAs/InGaAs/InAlAs Heterostructures

**DOI:** 10.3390/s23031441

**Published:** 2023-01-28

**Authors:** Algirdas Sužiedėlis, Steponas Ašmontas, Jonas Gradauskas, Aurimas Čerškus, Karolis Požela, Maksimas Anbinderis

**Affiliations:** 1 Center for Physical Sciences and Technology, Saulėtekio Ave. 3, LT-10257 Vilnius, Lithuania; 2 Vilnius Gediminas Technical University, Saulėtekio Ave. 11, LT-10223 Vilnius, Lithuania

**Keywords:** microwave detection, voltage sensitivity, bow-tie microwave diode, indium gallium arsenide, electromotive force of hot carriers, photo-gradient electromotive force

## Abstract

The application of the unique properties of terahertz radiation is increasingly needed in sensors, especially in those operating at room temperature without an external bias voltage. Bow-tie microwave diodes on the base of InGaAs semiconductor structures meet these requirements. These diodes operate on the basis of free-carrier heating in microwave electric fields, which allows for the use of such sensors in millimeter- and submillimeter-wavelength ranges. However, there still exists some uncertainty concerning the origin of the voltage detected across these diodes. This work provides a more detailed analysis of the detection mechanisms in InAlAs/InGaAs selectively doped bow-tie-shaped semiconductor structures. The influence of the InAs inserts in the InGaAs layer is investigated under various illumination and temperature conditions. A study of the voltage–power characteristics, the voltage sensitivity dependence on frequency in the K_a_ range, temperature dependence of the detected voltage and its relaxation characteristics lead to the conclusion that a photo-gradient electromotive force arises in bow-tie diodes under simultaneous light illumination and microwave radiation.

## 1. Introduction

The terahertz (THz) region of electromagnetic radiation is attractive for various applications due to its unique properties, such as its ability to penetrate through thin layers of nonconductive materials and its ability to be absorbed by water. The applications of THz radiation in imaging range from quality control to security screening [1]. Terahertz spectroscopy possesses a significant potential in biomedical research because the radiation excites low-frequency molecular vibrations, which can be successfully used in tissue detection, for example, to distinguish between pathological tissue and normal tissue [2]. The THz frequency band is also expected to be a huge resource for future wireless communications in 6G networks [3]. Both the sources and sensors of THz radiation are important components in the development of this promising technology. Schottky barrier diodes (SBDs) are the most common matured elements in the detection of THz radiation at room temperature [4,5]. Although SBDs have excellent properties, such as a high voltage sensitivity, low noise, a wide-frequency bandwidth and a high reliability, they suffer from a strong dependence on the process of Schottky junction formation and environmental conditions because the junction is formed on a semiconductor surface. Therefore, the search for new THz radiation sensors remains a topical issue. The need for new THz sensors that operate without cryogenic cooling and without external bias brings new challenges to the scientific and engineering community. The room-temperature detection of THz radiation by using plasma-wave resonance and wide-band non-resonant effects in various field-effect transistor formations on the basis of two-dimensional [6,7,8] and one-dimensional [9,10] structures has been reported. A ballistic rectifier with an artificial, asymmetric, selectively doped semiconductor scatterer has been proposed to detect microwave radiation at both cryogenic [11] and room [12] temperatures. Graphene-based ballistic rectifiers have been successfully used for detection and imaging in the THz frequency range [13,14]. A nanometer-scale self-switching device (SSD) has shown a nonlinear diode-like I-V characteristic in a barrier-less structure [15]. The arrays of SSDs containing selectively doped semiconductor structures have been used to detect electromagnetic radiation in sub-THz [16,17] and THz [18] frequency ranges at room temperature. Graphene SSDs have also revealed themselves to be room-temperature zero-bias sensors of electromagnetic radiation: in previous studies, an array of nine graphene SSDs was used to detect microwave radiation of up to 67 GHz and was expected to have a potential ability to detect terahertz radiation up to 1.5 THz [19], and a graphene SSD bridge rectifier was found to able to detect radiation up to 0.56 THz [20].

In a previous study, a nonuniform distribution of the electric field was built in homogeneous asymmetrically necked semiconductor samples [21]. Thus, another barrier-less semiconductor structure was proposed for detecting electromagnetic radiation, the so-called bigradient diode [22]. Later, the room-temperature zero-bias detection of wide-frequency-range electromagnetic radiation was demonstrated using asymmetrically shaped semiconductor structures, the so-called planar bow-tie microwave diodes with *n-n^+^* junctions in the narrowest part of the diode [23]. The symmetrical bow-tie configuration of the device is used in various applications; for example, this configuration is used in switchable antenna systems for bi-directional sensor applications [24], and it is used to control silicon-based dielectric nano-bow-tie dimers and to manipulate nanometer-scaled objects [25]. The principle of the operation of microwave bow-tie diodes is based on non-uniform carrier heating in a microwave electric field due to the broken geometrical symmetry of the diode, as well as the specific doping profile of the structure. This operational principle determines nearly frequency-independent voltage sensitivity values within the 10 GHz to 0.7 THz range for planar microwave diodes fabricated on the bases of thick GaAs epitaxial layers [26] and for those fabricated on the base of two-dimensional selectively doped GaAs/AlGaAs semiconductor structures [27]. Asymmetrical microwave bow-tie diodes with *n-n^+^* GaAs junctions have revealed themselves to be diodes that are more sensitive than the analogous microwave diodes of the symmetrical bow-tie configuration [28]. The bow-tie diodes on the bases of InGaAs semiconductor structures have been used for the heterodyne [29], spectroscopic [30] and homodyne spectroscopic [31] imaging of concealed objects. The polarity of the voltage induced across the ends of asymmetrically shaped bow-tie Si [23] and GaAs [26] diodes containing *n-n^+^* junctions has been found to correspond to the polarity of the electromotive force of hot electrons across the *n-n^+^* homo-junction. However, the origin of the voltage induced across bow-tie diodes containing selectively doped semiconductor structures remains ambiguous. For example, there are no data about the polarity of the voltage detected across the terminals of InGaAs-based bow-tie diodes [29,30,31]. The wider application of InGaAs-based bow-tie diodes requires a more detailed understanding of the detection mechanisms. Therefore, microwave bow-tie diodes with a broken geometrical symmetry fabricated on the bases of selectively doped InAlAs/InGaAs semiconductor structures with and without InAs inserts are studied in this article. Moreover, the peculiarities of microwave-induced voltage are studied by adding visible light illumination as an additional instrument for analyses.

## 2. Samples and Measurement Techniques

A selectively doped InAlAs/InGaAs semiconductor heterostructure served as a base for the bow-tie diodes. The high electron mobility and saturated drift velocity in the channels of InAlAs/InGaAs/InAlAs heterostructures make this formation attractive for developing field-effect transistors operating in the millimeter-wavelength range [32]. High-electron-mobility transistors have also been used for terahertz generation [33] and detection [34]. The uncovered peculiarities of the electron interactions with polar optical and interfacial phonons in semiconductor quantum wells [35] opened the possibility of controlling the electric and photoelectric properties of semiconductor heterostructures by using electron–phonon scattering engineering when electrons and phonons are confined by the insertion of phonon walls in a quantum well [36]. Electron mobility was found to experimentally increase when an InAs phonon wall was introduced into an InAlAs/InGaAs quantum well and when a nanometer-wide GaAs layer was introduced into the InAlAs barriers close to the interface of the InGaAs quantum well [37].

Two types of selectively doped InAlAs/InGaAs heterostructures were designed for the fabrication of the bow-tie diodes: samples with InAs inserts (sample W) and samples without the inserts (sample WO). A cross-sectional view of the structures and their energy-band diagrams and electron density distributions are presented in Appendix A. The energy-band diagrams and electron distributions were calculated by solving the Poisson equation. The measured electrical parameters of the heterostructures (sheet electron density, mobility and sheet resistance *R_sh_*) are presented in Table 1.

**Figure 1:**
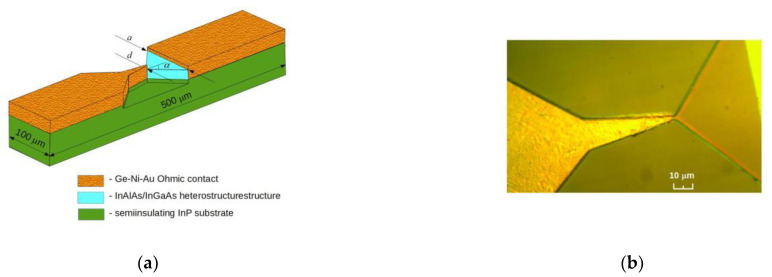
Schematic view (**a**) and microphotograph (**b**) of the planar bow-tie microwave diodes.

The electrical parameters of these two heterostructures were not much different. A slightly bigger difference can be seen in the figures presented in Appendix A: the electron density in the doping δ-layer of the WO structure was five times higher than that in the W heterostructure. A schematic picture and microphotograph of the planar bow-tie diodes are presented in Figure 1.

The details of the fabrication of the planar bow-tie microwave diodes are presented in [38]. A 60 nm etching depth was chosen to ensure the surefire confinement of the two-dimensional electron channels in the selectively doped semiconductor structures. The specific contact resistance and the sheet resistance of the conductive layer were measured using differently spaced ohmic contacts on a rectangular semiconductor mesa [39]. The values of the specific contact resistance *ρ_c_* of the bow-tie diodes and the sheet resistance *R_sh_* of the semiconductor structures are presented in Table 2. These were measured in the dark and under the illumination of visible light (see below). Two types of bow-tie diodes with 1 and 2 micrometer-wide “necks”, i.e., the narrowest part *d* (see Figure 1a), were fabricated.

High values of the specific contact resistance and strong scattering have substantial influences on the detected voltage magnitude and its distribution. The values of the measured sheet resistance were less scattered. However, as a result, the value of the electrical resistance of the diodes experienced substantial scattering.

All the measurements of the electrical parameters of the bow-tie diodes were performed using dc and high-frequency probe stations. The current–voltage (*I-V*) characteristics were measured using the Süss Micro Tec probe station EP6 with dc probes (FormFactor, Inc., Livermore, CA, USA) and Agilent E5270B Precision Measurement equipment (Agilent Technologies, Inc., Santa Clara, CA, USA). The voltage–power (*V-P*) characteristics of the diodes were measured in the K_a_ frequency range using a Cascade Microtech (FormFactor, Inc., Livermore, CA, USA) high-frequency probe station, and ACP40-A-GS-250 probes were used to connect the diodes to the measurement station. An SHF BT45 broadband bias tee separated the detected dc voltage signal from the microwave signal. The high-frequency measurement setup is presented in Appendix B [40]. The usage of the probe stations made the investigation more simple and allowed for on-wafer experiments to be performed under varying illumination conditions and at different temperatures. The microwave diodes were illuminated with the photo-lamp Eiko EKE21V150W (color temperature of 3240K). The temperature of the diodes in both the dc and high-frequency measurements was varied from room temperature up to 80°C using a commercially available Peltier modulus. The high-frequency measurements were performed in the K_a_ frequency range (26 ÷ 37.5 GHz). As a source of microwave radiation, a millimeter-wave sweep generator, G4403E (Elmika Ltd., Vilnius, Lithuania), on the base of the Hall transducer, was used.

## 3. Results and Discussion

The electrical resistance *R* of the bow-tie diodes is determined via the geometrical resistance *R_g_* of the active region and the contact resistance *R_c_* of the diode:

(1) $$R=R_{g}+R_{c}=\frac{R_{sh}}{2\tan\alpha }\ln\frac{a}{d}+\frac{\rho_{c}}{d}$$

where *R_sh_* is the sheet resistance of the selectively doped semiconductor structure; *a* and *d* indicate the width of the diode in its widest and narrowest parts, respectively; *α* notes the widening angle of the semiconductor structure (see Figure 1a); and *ρ_c_* stands for the specific contact resistance of the diode. Considering the exact sheet resistance values of the W and WO semiconductor structures and the scattered values of the specific contact resistance in Table 2, the possible electrical resistance range limits of the illuminated diodes were calculated using Equation (1). The results are presented in Table 3.

The statistical distribution of the electrical resistance is presented in Appendix C. The diodes with the InAs inserts, that is, the W-diodes, showed mean and median values that were the electrical resistance, while most of the WO-diodes had an electrical resistance that was higher than the mean value. Only the diodes with an electrical resistance within the presumed range were chosen for further investigation: 40 W-diodes out of 56 (71%) and 23 WO-diodes out of 47 (49%). It is worth noting that more diodes with a neck width *d* = 1 µm fell within the expected resistance range: 23 (compared to 17 diodes with *d* = 2 µm) in the case of the W structure and 14 (compared to 9 diodes with *d* = 2 µm) in the case of the WO structure. This experimental fact could be explained by the in-plane inhomogeneity of the semiconductor structure.

**Figure 2:**
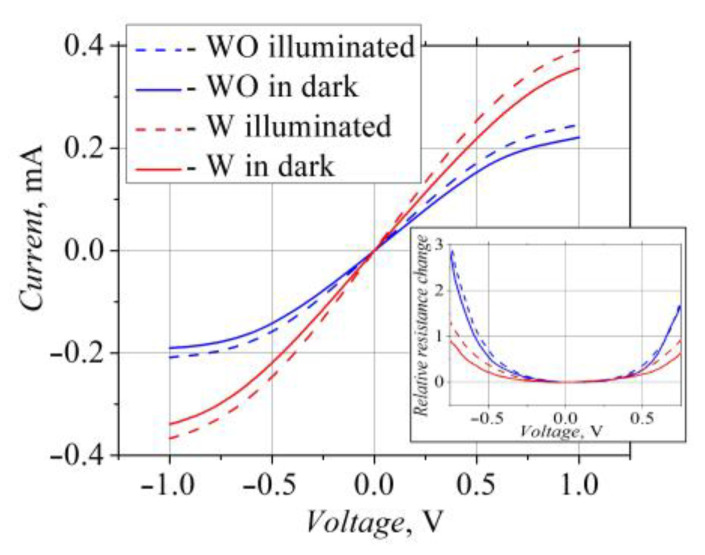
Current–voltage characteristics of the bow-tie diodes in the dark (solid lines) and under illumination (dashed lines, illuminance 14,400 lx.). In the inset: dependences of the relative resistance change on applied voltage.

The electrical properties of the bow-tie diodes were sensitive to the visible light illumination. The electrical resistance of the diodes, both the W and WO types, decreased by approximately 20% under the action of the light. The *I-V* characteristics of the bow-tie diodes were sublinear (Figure 2). For more details, the inset of Figure 2 shows the dependence of the relative resistance change $\Delta R=\frac{R\left(U\right)-R\left(0\right)}{R\left(0\right)}$ on the voltage applied across the diodes in the dark and under illumination. The dependence of the electrical resistance on the voltage was more expressed in the case of the WO-diodes, while the W-diodes were more sensitive to illumination.

**Figure 3:**
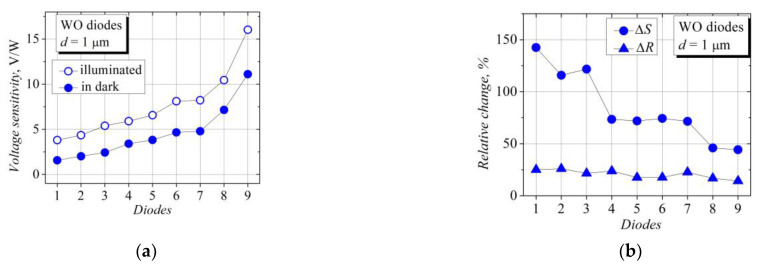
(**a**) Statistical representation of voltage sensitivity of some bow-tie diodes on the base of WO structures under illumination (open dots, illuminance 14,400 lx.) and in the dark (solid dots); (**b**) statistical representation of illumination-induced relative change in voltage sensitivity Δ*S* and electrical resistance Δ*R* of the same WO-diodes.

However, the detection properties of the bow-tie diodes depended on the illumination in a different way. First, the polarity of the voltage detected across the WO-diodes corresponded to the polarity of the Schottky diode-detected voltage; i.e., a negative potential arose on the narrower part of the bow-tie structure (left side of the diode in Figure 1). This diode terminal was grounded during the measurements, so the sign of the detected voltage was positive. We called the detected voltage of this polarity the Schottky voltage and further denoted it as the SCH voltage, and it is displayed in the graphs as a positive voltage. In the case of illumination, approximately half of the W-diodes detected a voltage of the opposite polarity; i.e., a positive potential arose on the narrower part of the bow-tie structure. We called this polarity voltage the thermoelectromotive force voltage, the TEMF voltage, and it is displayed in the graphs as a negative voltage. When the bow-tie WO-diodes were placed into the microwave electric field, the illumination increased the detected voltage value while keeping its polarity the same. The influence of the illumination on the voltage sensitivity of the nine WO-diodes can be seen in Figure 3a; here, the diodes are lined up in ascending order with regard to their voltage sensitivity.

**Figure 4:**
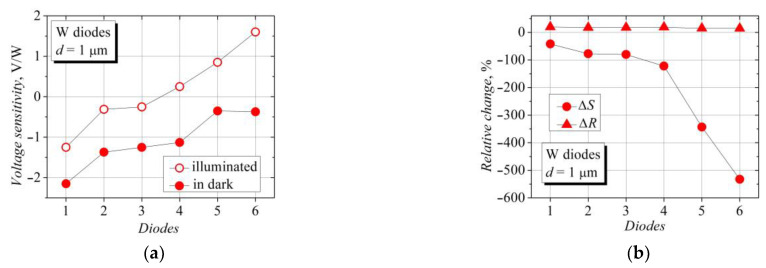
(**a**) Statistical representation of voltage sensitivity of six bow-tie diodes on the bases of W structures under illumination (open dots, illuminance 14,400 lx.) and in the dark (solid dots); (**b**) statistical representation of illumination-induced relative change in voltage sensitivity Δ*S* and electrical resistance Δ*R* of the same W-diodes.

The voltage sensitivity of the diodes *S* is expressed as

(2) $$S=\frac{U_{d}}{P}$$

where *U_d_* notes the detected voltage, and *P* is the incident microwave power. Figure 3b depicts the relative change in the voltage sensitivity and the electrical resistance of the same nine WO-diodes due to the illumination. The relative percentage changes in the voltage sensitivity Δ*S* and the electrical resistance Δ*R* of the bow-tie diodes are determined as follows:

(3) $$\Delta S=100\cdot \frac{S_{ill}-S_{dark}}{S_{dark}},\;\;\;\;\;\Delta R=100\cdot \frac{R_{dark}-R_{ill}}{R_{dark}},$$

where the subscripts “*ill*” and “*dark*” denote the parameters of the diodes under illumination and in the dark, respectively. The effect of the illumination on the voltage sensitivity and the electrical resistance of the six W-diodes is shown in Figure 4. The illumination reduces the electrical resistance of the diodes, in the cases of both the W and WO types, and it increases the voltage sensitivity of the WO-diodes. However, different behavior is observed in the case of the W-diodes. Some W-diodes show a decrease in the detected voltage value, and out of these, those with a lower sensitivity change their voltage polarity from the TEMF to the SCH sign under the action of the illumination (see Figure 4a). The relative change in the electrical resistance is scattered between 15 and 25 percent, while the relative change in the voltage sensitivity is much more significant and reaches hundreds of percent.

**Figure 5:**
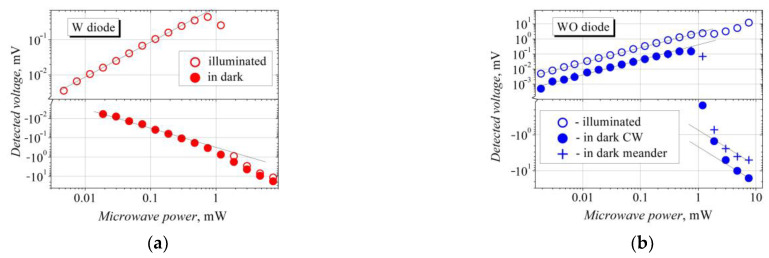
Voltage–power characteristics of the bow-tie diodes on the bases of W (**a**) and WO (**b**) semiconductor structures in the dark (solid dots) and under white-light illumination. The illuminance of the lamp was 14,400 lx. The lines are guides of linear dependence.

The voltage–power characteristics of the bow-tie diodes based on the W structure are presented in Figure 5a. A linear dependence of the detected voltage can be seen at a low microwave power. The super-linearity of the *V-P* characteristic begins in the dark at an intense microwave radiation, and this change can be caused by the substantial heating of the semiconductor crystal lattice via continuous-wave (CW) microwave radiation. An ordinary lattice-heating-induced thermal electromotive force arises, and it contributes to the thermal electromotive force of hot electrons, thus making the voltage–power characteristic super-linear. In the case of illumination, the linear *V-P* characteristic of the W-diode turns into a sublinear one at a higher incident microwave power. This sublinearity and further change in the detected voltage polarity may also be caused by the lattice-attributed thermal electromotive force and by the appearance of the negative differential resistance in the semiconductor due to the Gunn effect [41]. Here, the detected TEMF voltage adds to the SCH voltage of the opposite polarity, resulting in a decrease in the total detected voltage and even in the change in its polarity at a microwave power exceeding 1 mW. Note that the “dark” and the “illuminated” detected voltages become almost equal in magnitude when the maximum microwave power is applied (see Figure 5a).

The bow-tie diodes containing no InAs inserts, that is, the WO-diodes, generate the SCH voltage at a low microwave radiation power (see Figure 5b). However, as the power is increased, the “dark” *V-P* characteristic deviates from the linear law, and the detected voltage changes its polarity from the SCH to the TEMF sign. As the maximum microwave power is applied (slightly below 10 mW), the *V-P* characteristic, again, turns into its linear form, indicating the presence of an ordinary thermal electromotive force and the Gunn effect. When the meander-modulated microwave signal is applied to the WO-diode, the lattice-heating effect is minimized, and the contribution of the TEMF voltage to the total detected voltage becomes weaker.

**Figure 6:**
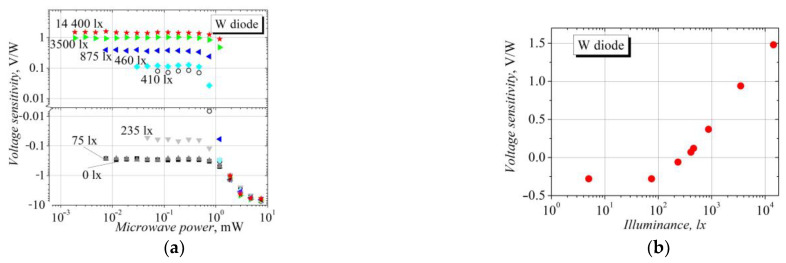
(**a**) Dependence of voltage sensitivity of the W-diode on incident microwave power at different white-light illuminance values. (**b**) Dependence of voltage sensitivity on illuminance at low microwave power.

The influence of the illumination on the voltage sensitivity of the W-diode can be seen in Figure 6. The voltage sensitivity versus the microwave power at various illuminance values is presented in Figure 6a. The stronger the light intensity, the lower the voltage sensitivity resulting from the TEMF polarity voltage within the linear *V-P* characteristic region; the sensitivity drops down to zero at approximately 300 lx (see Figure 6b for the negative values of sensitivity). A further increase in the light intensity turns the polarity of the detected voltage into the SCH one, and then the sensitivity increases sublinearly with the illuminance (Figure 6b). The range of the linear dependence of the detected SCH voltage also increases with the illuminance; i.e., the dynamic range of the power-independent voltage sensitivity widens. As mentioned above, a strong microwave radiation heats the semiconductor crystal lattice, and the thermal electromotive force arises across the ends of the diode; in this case, the illumination obviously has a weak influence on the detection properties of the bow-tie diode on the base of the InGaAs semiconductor structure with the InAs inserts (see the curves in Figure 6a demonstrating the voltage sensitivity at high microwave radiation power).

**Figure 7:**
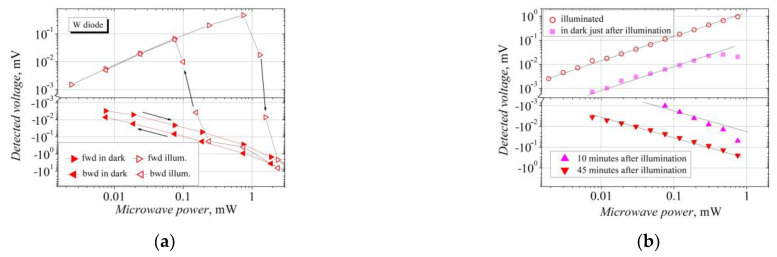
Demonstration of the detection inertness in the W bow-tie diodes: (**a**) influence of microwave power and (**b**) influence of 14,400 lx illumination. Arrows in (**a**) mark the direction of the measurement: fwd, when the microwave power is increased, and bwd, when the power is decreased. The lines in (**b**) are guides representing the linear law.

Another observable feature of the W-diodes is the inertness of their response to the microwave radiation. Different voltage–power characteristics of the W-diodes are observed with the microwave power rising to a maximum value (forward case—fwd) and with it declining to a minimum one (backward case—bwd). This inertness is not expressed during the measurements in the dark; however, the hysteresis of the *V-P* characteristic can be seen under illumination. As Figure 7a shows, the change in the voltage polarity occurs at a lower power value in the backward case. This discrepancy in the *V-P* characteristic can be explained by the crystal lattice heating under the action of the microwave radiation. In the bwd case, after the application of the maximum microwave power, some time is needed for a crystal lattice to cool down and to eliminate or diminish the contribution of the TEMF voltage to the total detected voltage. The voltage–power characteristics of the W-diode presented in Figure 7b were measured in the dark (square dots), under illumination (open circles) and in the dark 10 min (upward triangles) and 45 min (downward triangles) after illumination. To avoid the influence of the crystal lattice heating, the measurements were carried out in the linear region of the *V-P* characteristic, i.e., at low power values. As a result, the linear *V-P* characteristics are presented for measurements made in the dark and under illumination.

**Figure 8:**
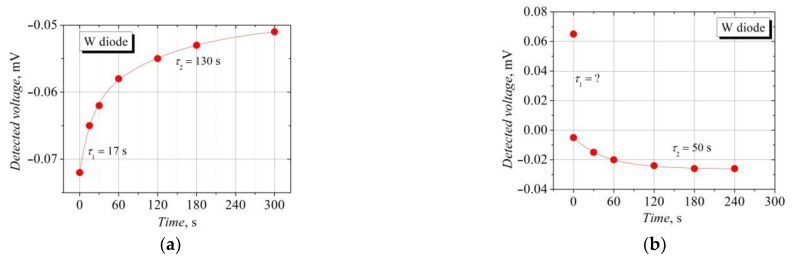
The relaxation of the voltage detected across the W-diode after it was affected by a strong microwave electric field (**a**) and after its illumination with visible light (**b**). Lines indicate approximation via exponential functions.

The time variation of the detected voltage across the W-diodes after they were exposed to a strong microwave electric field and illumination was worth a more detailed analysis. A low microwave power (~70 μW) was applied to avoid the heating of the diode during the measurements. First, the diode was affected by a strong microwave electric field of 7 mW power radiation for a couple of minutes. Then, the power was reduced by two orders of magnitude, and the detected voltage was measured at several time intervals. The measurements were carried out in the dark. The dependence of the detected voltage on time after the impact of the strong electric field is presented in Figure 8a. The induced voltage of the TEMF polarity relaxes to a stationary value that corresponds to the voltage sensitivity in the linear region of the voltage–power characteristic. The relaxation is approximated by an exponential function with two relaxation time constants: the relaxation starts with a time constant *τ_1_* = 17 s, which can be related to the crystal lattice cooling in the active region of the W-diode. A slower relaxation of the detected voltage with a time constant *τ_2_* = 130 s is unaccountable and requires further investigation. The relaxation of the detected voltage after illumination with a visible light is demonstrated in Figure 8b. A fast decrease in the detected SCH voltage and change in its polarity to the opposite TEMF one occurs just after switching the illumination off. The measurement capabilities did not allow us to estimate the time constant of this fast relaxation, so only the TEMF voltage relaxation was approximated, and this was carried out via an exponential function with a single relaxation time (*τ_2_* = 50 s). The long relaxation times of the detected voltage ranging tens of seconds are related to temperature-dependent phenomena.

**Figure 9:**
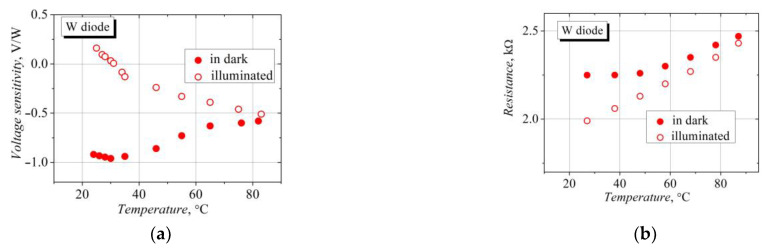
Temperature dependence of voltage sensitivity (**a**) and electrical resistance (**b**) of the bow-tie diodes on the base of a selectively doped InAlAs/InGaAs semiconductor structure with InAs inserts in the dark and under 14,400 lx illumination.

The dependence of the W-diodes’ voltage sensitivity on temperature is different in the dark and under illumination (Figure 9a). In the dark, the sensitivity corresponding to the TEMF polarity decreases non-monotonically with an increase in the temperature. This decline can be explained by the decrease in electron mobility and electron energy relaxation time in InGaAs with the increase in the temperature [42,43]. When the W-diode is illuminated, the detected SCH voltage decreases with the increase in the temperature, and further, the total voltage changes its polarity due to the domination of the thermal electromotive force at higher crystal lattice temperatures. The reduction in the hot-electron TEMF input in the dark due to the lattice heating and the increase in the ordinary TEMF of the illuminated and heated diode lead to the convergence of the “dark” and “illuminated” detected voltages, as can be seen in Figure 9a.

Illumination also influences the dependence of the electrical resistance of the W-diode on temperature (see Figure 9b). The resistance of the illuminated diode depends linearly on temperature, and it is almost constant at a temperature close to room temperature in the dark. At higher temperatures, the resistance of the “dark” diode begins to increase faster with temperature, and it approaches the resistance of the illuminated diode. The different temperature dependences can be explained as follows: the resistance of the illuminated diode is related to the dependence of the charge carrier mobility on temperature, while the resistance of the “dark” diode is additionally influenced by the change in the charge carrier density caused by the temperature change.

**Figure 10:**
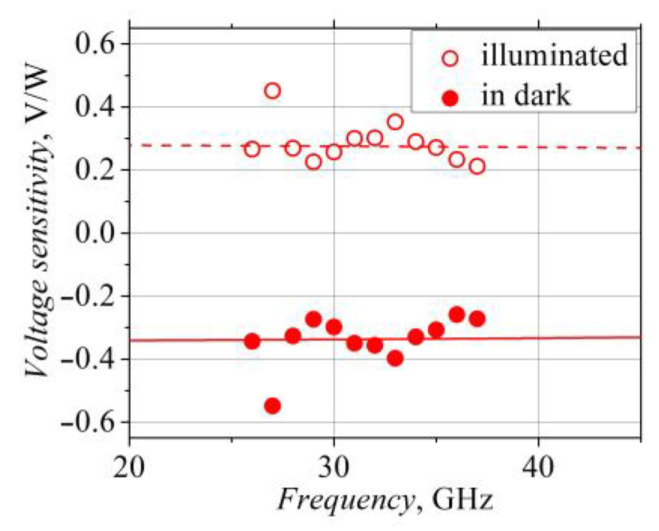
Frequency dependence of voltage sensitivity of the W-diode in the dark and under 14,400 lx illumination. The lines represent theoretical dependence of the voltage sensitivity on frequency in the case of bow-tie diode with semiconductor *n-n^+^* junction.

The dependence of the detected voltage on frequency can help better understand the origin of the voltage. These dependencies, in the dark and under illumination, are presented in Figure 10. They are similar; however, this is most probably not due to the properties of the W-diode itself but rather the features of the microwave signal transmitting tract, specifically the high-frequency probe station. The experimental frequency dependence of the voltage sensitivity of the W-diode in the dark can be compared with the theoretical voltage sensitivity of the bow-tie diode with the semiconductor *n-n^+^* junction [44]:

(4) $$S_{i}=\frac{U_{d}}{P_{i}}=\frac{2R_{sh}\mu_{0}\tan\alpha }{3d^{2}\ln\frac{a}{d}}\frac{P}{P_{i}}N,$$

where *P* is the microwave power absorbed by the diode; *P_i_* denotes the incident microwave power; *μ*_0_ is the low-field electron mobility; and *N* stands for the factor that depends on the frequency and electron energy, pulse and the Maxwell relaxation times [44]. The calculated frequency dependence of the voltage sensitivity of the W bow-tie diode with the electrical and geometrical parameters is presented in Figure 10 as a line. The frequency-independent voltage sensitivity of the illuminated W bow-tie diode in the K_a_ frequency range allows us to assume that the voltage detected by the illuminated diode is not caused by the contact phenomena but rather by the phenomena related to the charge carrier heating in the microwave electric field.

**Figure 11:**
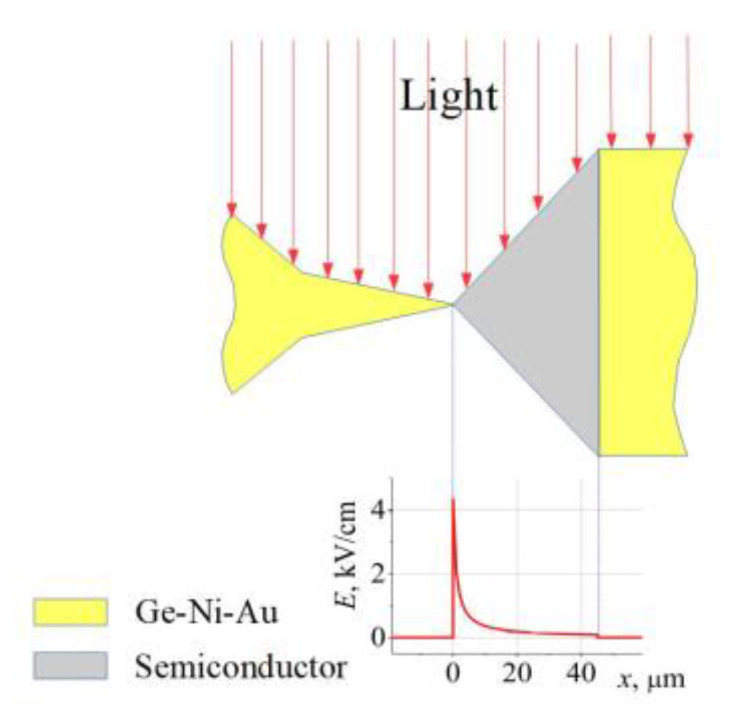
Schematic view of the bow-tie diode illuminated with light (**top**) and distribution of electric field in the active region of the diode with 1 V of applied voltage.

One of these phenomena is the photo-gradient effect of hot carriers [45]. The essence of the photo-gradient phenomenon lies in the rise of the electromotive force in light-illuminated homogeneous semiconductors when a large electric field gradient is created on one side of the illuminated region. The separation of the excess light-generated carriers occurs due to the Dember effect [46] and the different conditions of carrier diffusion in the semiconductor regions with a strong electric field and with no electric field. The light-illuminated bow-tie diode placed in a microwave electric field perfectly matches the conditions that give rise to the photo-gradient emf. A schematic view of the illuminated bow-tie diode is presented in Figure 11, and the electric field distribution in the bow-tie diode with an applied *U* = 1 V voltage was calculated as follows:

(5) $$E=\frac{2U}{(d+x\tan\alpha )\ln\frac{a}{d}},$$

where *x* denotes the longitudinal coordinate of the diode. The metallic Ge-Ni-Au contacts of the diode shield the semiconductor regions from illumination and reduce the electric field strength to zero. Therefore, the conditions that give rise to the photo-gradient emf are achieved since the electric field in the illuminated semiconductor structure is the strongest in its narrowest part and drops to zero at its widest side. The polarity of the photo-gradient emf is determined by the dependence of the diffusion coefficient of the charge carriers (electrons and holes) on the electric field strength. The photo-gradient emf is added to or subtracted from the thermal electromotive force of the hot carriers, which is also induced in the bow-tie diode under the action of the microwave electric field. In the case of the bow-tie diodes on the base of the InAlAs/InGaAs selectively doped structure with the InAs inserts, that is, the W-diodes, the photo-gradient emf exceeds the thermal emf of the hot carriers in the diode. Therefore, the polarity of the voltage detected across the W-diode corresponds to the TEMF polarity (in the dark), while the polarity of the voltage detected across the white-light-illuminated diode is in accordance with the SCH polarity that corresponds to the polarity of the photo-gradient emf. In the case of the WO-diodes fabricated on the base of InAlAs/InGaAs without InAs inserts, the voltage detected in the dark has an SCH polarity, and the illumination only slightly increases the total detected voltage due to the arising of the photo-gradient emf.

## 4. Conclusions

This study of the bow-tie diodes on the bases of InAlAs/InGaAs selectively doped semiconductor structures with and without InAs inserts in the InGaAs layer allowed us to draw the following conclusions:

1. The dc and high-frequency electrical properties of the bow-tie diodes, i.e., their electrical resistance and voltage sensitivity, were sensitive to the illumination of the diode with visible light.
2. The voltage sensitivity of the diodes with the InAs inserts was more responsive to the illumination.
3. When the bow-tie diodes with the InAs inserts with a typical lower voltage sensitivity were affected by the visible light, the polarity of the voltage detected across them changed from that of a thermoelectric electromotive force of hot carriers to the opposite one.
4. The illumination increased the voltage sensitivity of the bow-tie diodes that had no InAs inserts.
5. Analyses of the voltage–power characteristics, frequency dependence of the voltage sensitivity in the K_a_ frequency range, the temperature dependence of the detected voltage and the dynamics of the detected voltage through time allowed us to assume that the photo-gradient electromotive force arose in the bow-tie diodes under illumination.
6. The visible-light-induced increase in the photo-gradient emf changed the polarity of the detected voltage in the bow-tie diodes with the InAs inserts and increased the magnitude of the detected voltage in the diodes without the inserts.

## Figures and Tables

**Table 1:** Electrical parameters of the grown semiconductor layered structures.

Heterostructure	Sheet Density *n_sh_*, cm^−2^	Electron Mobility *μ*, cm^2^/(V·s)	Sheet Resistance *R_sh_*, Ω/☐
W	1.52 × 10^12^	7760	530
WO	1.39 × 10^12^	8390	536

**Table 2:** Specific contact resistance of the bow-tie diodes and sheet resistance of the selectively doped W and WO semiconductor structures in the dark and under visible light illumination.

Bow-Tie Diodes on the Base of	In the Dark		Illuminated	
	*ρ_c-d_*, Ω·mm	*R_sh-d_*_,_ Ω/☐	*ρ_c-ill_*, Ω·mm	*R_sh-ill_*_,_ Ω/☐
W heterostructure	1.55 ± 1.01	530 ± 50	1.33 ± 0.77	462 ± 39
WO heterostructure	1.29 ± 0.60	542 ± 50	1.25 ± 0.64	479 ± 39

**Table 3:** Presumed range of electrical resistance of the illuminated bow-tie diodes.

Semiconductor Heterostructure	Width *d*, μm	Presumed Electrical Resistance, kΩ
W	1	1.54 ÷ 3.08
	2	1.12 ÷ 1.89
WO	1	1.62 ÷ 2.90
	2	1.17 ÷ 1.81

**Figure A1:**
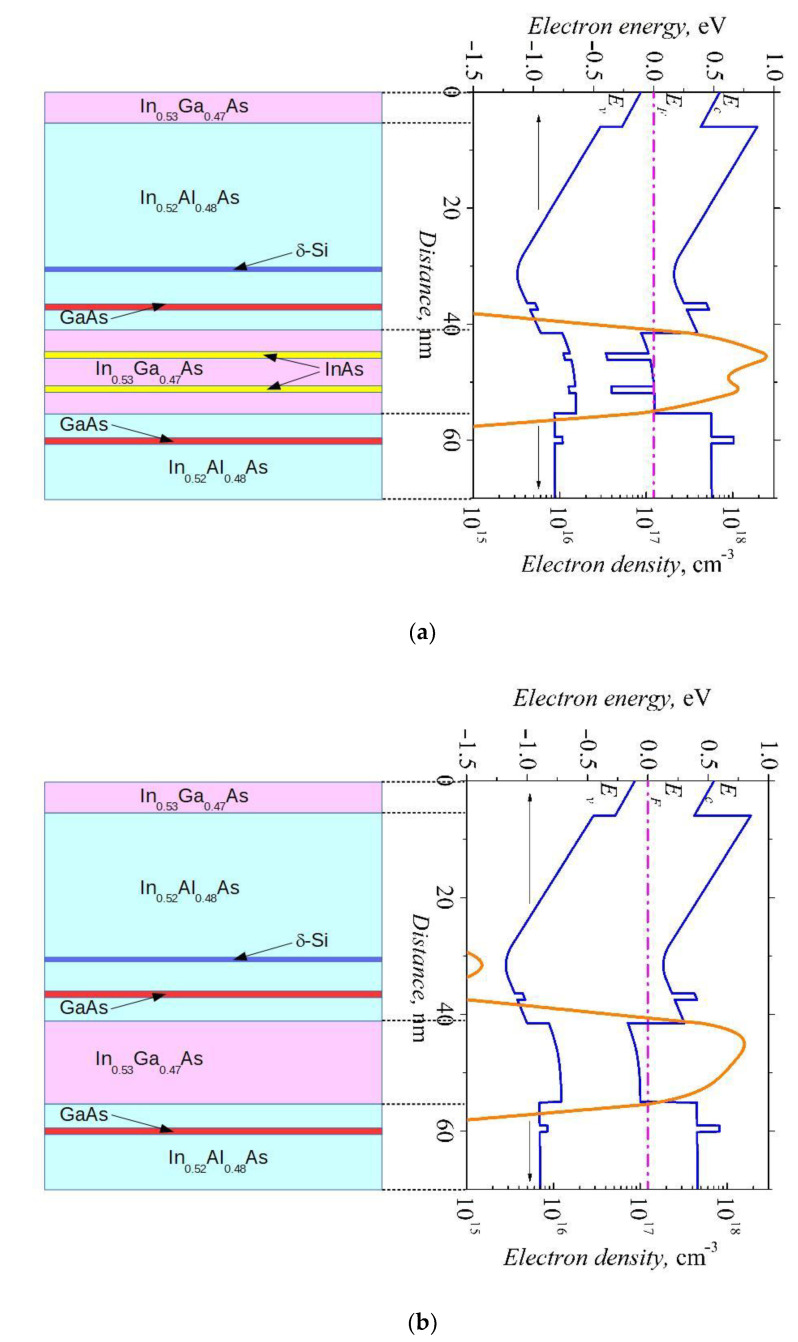
(**a**) The W heterostructure with the InAs inserts. (**b**) The WO heterostructure without the InAs inserts.

**Figure A2:**
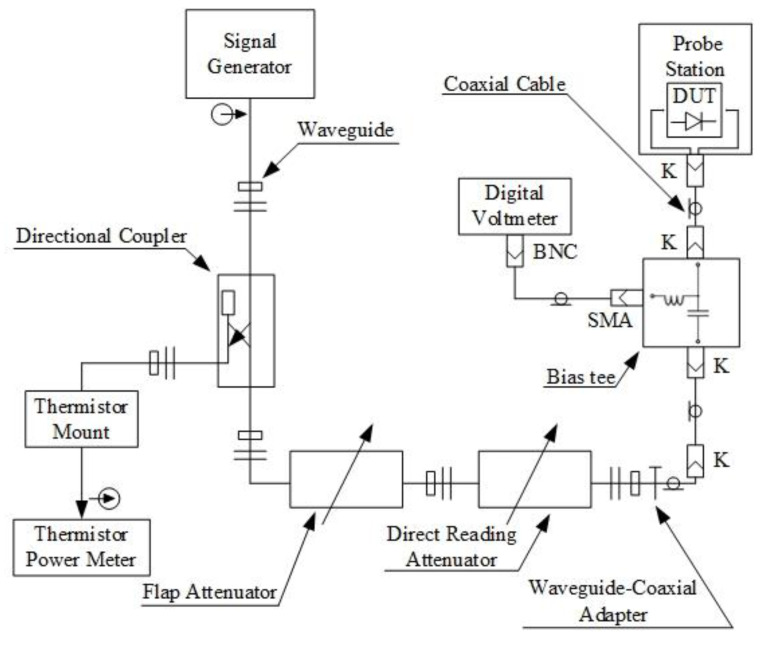
The structure block diagram of the measurement setup.

**Figure A3:**
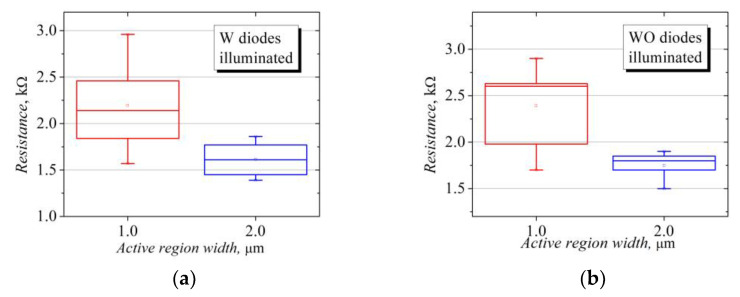
(**a**) The W bow-tie diodes with InAs inserts; (**b**) the WO bow-tie diodes without InAs inserts.

## Data Availability

Data sharing is not applicable to this article.

## Appendix A

Cross-section of the investigated semiconductor structures and their energy-band diagrams with electron density distributions. The thicknesses of the GaAs and InAs inserts are 2.4 nm and 1.2 nm, respectively. The thickness of the δ-Si doped layer is 2 nm.

## Appendix B

A structure block diagram of the probe station for the measurement of the detected voltage dependencies on microwave power and frequency in the K_a_ frequency range [40].

The measurement setup involves a TWT signal generator, “Elmika G4408E”, working in the CW mode; coaxial-waveguide adapters, “Elmika CWA-08/K(f)”; a WR28 waveguide transmission line; an “Elmika” directional coupler; a thermistor mount, “M5-45”; a thermistor power meter, “M3-22A”; a flap waveguide attenuator; a direct-reading attenuator; a broadband bias tee, “SHF BT 45 B”; a “Cascade Microtech EP6” probe station with air coplanar probes, “ACP 40 A GS 250”; a digital voltmeter, “Agilent 34401A”; and connecting cables.

## Appendix C

A statistical presentation of the electrical resistance of the bow-tie diodes under illumination with a visible light of 14,400 lx.
